# Chronic Migraine Resistance: A Case Report Highlights an Overlooked Factors in Family Medicine Clinics

**DOI:** 10.3390/healthcare14131874

**Published:** 2026-06-26

**Authors:** Maram H. Alshareef

**Affiliations:** Department of Community Medicine and Pilgrims Health Care, College of Medicine, Umm Al-Qura University, Makkah 21955, Saudi Arabia; mhashareef@uqu.edu.sa

**Keywords:** medication overuse headache, chronic migraine, multidisciplinary approach, prevention, stress management, triptan overuse

## Abstract

**Highlights:**

**What are the main findings?**

- Medication overuse can mimic treatment-resistant migraine.

**What are the implications of the main findings?**

- Chronic migraine might be prevented through early screening for medication-overuse headache, initiation of proper prophylaxis, structured patient education, stress management, and strict regulation of over-the-counter triptan dispensing.

**Abstract:**

**Background:** Chronic migraine may seem treatment-resistant when underlying medication-overuse headache (MOH) and psychosocial stressors are overlooked. This report describes a patient who was seen in a family medicine clinic with persistent triptan overuse and daily social stressors concealing the cause of her chronic headaches and highlights how pharmacologic and behavioral strategies restored control. **Case presentation:** A woman in her 40s with a 10-year history of migraine developed chronic daily headaches after years of near-daily eletriptan use. This persistent triptan overuse led to the failure of multiple preventive therapies during ongoing overuse. Symptom remission after eletriptan withdrawal confirmed the diagnosis of MOH. Subsequent stabilization with preventive treatment encouraging self-management and behavioral and physiotherapy interventions allowed her to achieve sustained remission and regain functional stability. **Conclusions:** This case demonstrates that unrecognized medication overuse, combined with stress and unrestricted access to triptans, can mimic treatment-resistant migraine. Chronic migraine can be managed and prevented through early screening for MOH, structured patient education, multidisciplinary management approaches, and strict regulation of OTC triptan dispensing.

## 1. Background

Chronic migraine has a prevalence of 1–2% in the general population. It is a progressive condition that can develop over time, with an annual transition rate of about 2–3% from episodic to chronic migraine [1]. Several modifiable factors have been identified, and early recognition of these plays a crucial role in preventing this progression. Timely diagnosis and appropriate management can therefore reduce the risk of chronicity and its associated burden [2]. Migraine is a complex neurological disorder involving both peripheral and central pain pathways. The transition from episodic to chronic migraine is thought to result from repeated activation of the trigeminovascular system leading to peripheral and central sensitization, cortical hyperexcitability, and an altered pain process. Several modifiable factors, including medication overuse, psychosocial stress, sleep disturbances, and psychiatric comorbidities, have been associated with migraine chronification. In the present case, prolonged medication overuse and ongoing psychosocial stress may have contributed to the persistence of chronic migraine, highlighting the importance of early recognition and management of these risk factors [3,4]. Chronic migraine is characterized by headaches occurring for ≥15 days per month, with ≥8 days featuring migraine characteristics, such as throbbing pain, nausea, sensitivity to light and sound, or visual disturbances [5]. Managing acute migraine attacks requires abortive medications—including triptan, non-steroidal anti-inflammatory drugs, and newer medications such as gepants—to relieve symptoms [6]. Eletriptan, a selective 5-HT1B/1D receptor agonist, is effective; however, its frequent use can cause medication-overuse headache (MOH) [7]. Triptans are not associated with substance use disorder because they do not produce euphoric effects or typical reinforcing behaviors. However, overuse can cause rebound headaches via neuronal hyperexcitability and trigeminal sensitization [8]. Timely diagnosis and initiation of prophylaxis had been proven and used to treat chronic migraine [5]. Gepants represent a newer class of medications effective for both acute and preventive migraine treatment. Unlike triptans, they improve the treatment response while reducing the risk of rebound headaches associated with MOH [9]. This distinction is particularly important, given that MOH is a chronic disorder defined by headaches occurring for ≥15 days per month, with overuse of acute medications (≥10 days per month) for >3 months [10].

Despite significant advances in the management of chronic migraine, many patients are underdiagnosed and undertreated. In primary care, important contributing factors such as MOH, psychosocial stress and psychiatric comorbidities are often overlooked, which can delay diagnosis and limit the effectiveness of treatment [2]. MOH is one of the most frequent and preventable causes of treatment resistance, and chronic migraine may appear refractory when factors such as medication overuse and psychosocial stress are overlooked [11,12]. This case report illustrates how persistent triptan use and unrecognized stress perpetuated headache chronicity, and how an integrated pharmacological–behavioral approach achieved complete remission. In addition, increasing evidence suggests that these patients frequently present with musculoskeletal dysfunctions, including cervical impairments, temporomandibular dysfunction, myofascial pain, and altered pain processing. Addressing these dysfunctions through physiotherapy and multimodal rehabilitation may contribute to reducing headache frequency, improving disability, and potentially decreasing medication overuse [13,14]. This case highlights the important role of family physicians in identifying and addressing chronic migraine early in its course. In addition, it illustrates how patient-centered care and tailored treatment strategies can improve outcomes, enhance quality of life, and reduce the overall burden of the disease on both patients and healthcare services [15,16].

## 2. Case Presentation

This case involved a female patient in her forties with a bachelor’s degree in computer science. She is married, a housewife and a mother of three sons, with the oldest aged 16 and the youngest 5 years. She presented to a family medicine clinic with a 10-year history of migraine, which progressed to chronic daily headache over the past 10 years. Her headaches were predominantly right-sided, occasionally left-sided or generalized. The pain was described as throbbing and was associated with photophobia, phonophobia, and nausea. She reported approximately more than eight migraine days per month requiring rest in a dark, quiet room. In addition, she experienced a separate dull aching daily, a generalized headache of stable severity over several years, which significantly interfered with daily household activities. The headache severity remained the same over the years, with no change in pattern. Triptans were reported as the only effective abortive therapy.

The patient denied focal neurological symptoms, including weakness, numbness, or speech disturbance, and denied systemic symptoms such as fever, weight loss, or anorexia. Her past medical history was significant for hypertension, treated with irbesartan 300 mg daily for five years, and anxiety diagnosed four years prior to presentation, recently managed with escitalopram with good control. She reported significant psychosocial stress related to childcare and household responsibilities. Her caffeine intake was limited to one to two cups of coffee daily. She denied the use of other analgesics, alcohol, or illicit substances.

Her family history was positive for migraine in multiple first- and second-degree relatives. A neurological examination was unremarkable, with normal cranial nerve function, motor strength, sensation, coordination, and gait examination. Bilateral masseter muscle tenderness was noted, consistent with stress-related temporomandibular joint involvement. An ophthalmological examination, including a visual acuity assessment and dilated fundoscopic examination, was normal, with no evidence of papilledema or optic disc abnormalities. A brain Magnetic Resonance Imaging (MRI) study with contrast revealed no abnormalities. Laboratory investigations, including a complete blood count, liver function tests, renal function tests, thyroid-stimulating hormone levels, the erythrocyte sedimentation rate, and c-reactive protein, were within normal limits.

### 2.1. Treatment

Prior to presentation, she was treated with propranolol (20 mg) twice daily for 4 years. The treatment initially provided good headache control, but its efficacy appeared to decline during periods of increased caregiving and household responsibilities, particularly during the school year. These demands included transporting her children to school, managing household tasks, and maintaining routine family activities, which caused sleep deprivation. The patient reported that these increased responsibilities were associated with worsening headache control despite continued treatment. A trial to increase the dose caused fatigability and dizziness; in addition, it affected her blood pressure negatively. Further medication history revealed that she was prescribed escitalopram (20 mg daily), which improved the stress but not the headache severity. During follow-up, the patient’s treatment regimen was modified by stopping propranolol, gradually tapering escitalopram, and initiating low-dose nightly amitriptyline (10 mg, followed by 25 mg) for headache prophylaxis and anxiety symptom management; however, it failed to even partially affect her headache frequency and caused side effects of weight gain and sleepiness that affected her daily activities. In addition, it did not control her anxiety much, so it was stopped and she went back to escitalopram. Simultaneously, she was consuming over-the-counter (OTC) eletriptan (40 mg almost daily) without guidance, taking it every day. A subsequent trial of topiramate (25 mg twice daily) was poorly tolerated due to a side effect of finger tingling; subsequently, the dose could not be escalated. The headache frequency remained unchanged. She later received botulinum toxin A injections (150 IU every 12 weeks × 3 cycles), without benefit.

Unsupervised daily triptan use continued and was not addressed by the treating doctor. Later, Erenumab (Aimovig, 70 mg monthly) was initiated. This regimen remained ineffective until eletriptan became temporarily unavailable, after which her headaches resolved within weeks and remained absent for 6 months—confirming MOH as the cause of refractory headache poorly responding to proven prophylaxis medication for chronic migraine.

### 2.2. Outcome and Follow-Up

Six months later, eletriptan was restocked. Under the burden of ongoing daily stress, the patient resumed taking triptans in an attempt to manage and cope with her headache symptoms, and the daily headaches recurred. Although her physician limited the prescription, she still easily accessed the drug OTC. She described triptan as “the only thing that partially relieved the pain and allowed me to function” when her preventive medication was unavailable.

Atogepant (Aquipta, 60 mg once daily) was initiated as a preventive therapy; the patient showed a better response which showed results later. At the 3-month follow-up, she demonstrated improved headache control, with a reduction in attack frequency to approximately two episodes per month and a marked decrease in the use of eletriptan. In addition, Ubrogepant (100 mg) was added as an abortive medication with instructions about its daily and monthly frequency. This regimen seemed to work better when escitalopram (20 mg daily) was resumed for anxiety management. This combination eliminated her headaches and enabled complete triptan cessation (Table 1).

Three months later, during a period of temporary unavailability of atogepant, the patient experienced a relapse of daily chronic headache and self-administered eletriptan obtained over the counter. Following the restocking and reinitiation of atogepant and the introduction of structured stress-management interventions, including relaxation techniques such as diaphragmatic breathing exercises, guided mindfulness meditation, sleep-hygiene counseling and physiotherapy sessions focused on stretching and strengthening exercises for the neck and upper back muscles, the patient reported a gradual improvement in headache control [17,18]. The stress-management interventions were aimed at addressing the impact of ongoing psychosocial stressors, while physiotherapy targeted tension and postural factors that contribute to headache symptoms. Over subsequent follow-up visits, she experienced progressive improvement and achieved remission with sustained functional stability, which allowed her to maintain her usual daily activities without significant headache-related impairment.

## 3. Discussion and Conclusions

This case demonstrates how unrecognized medication overuse can cause treatment resistance. Various preventive trials failed while triptan overuse persisted [1]. Once eletriptan was withdrawn, the diagnosis of MOH became evident, and improvement was observed [19]. Several case reports have highlighted how triptan overuse can transform episodic migraine into chronic migraine and underscore the need for stricter prescribing oversight and patient education to prevent uncontrolled access and overuse.

The treatment of chronic migraine focuses on reducing headache frequency and severity and improving patients’ quality of life through combined acute and preventive strategies. Preventive therapies aim to reduce the number of attacks with medications, such as beta-blockers, anticonvulsants, antidepressants, and calcitonin gene-related peptide (CGRP) inhibitors [5]. Botulinum toxin type A is also effective, particularly for patients experiencing frequent attacks. Failure to withdraw from the medication overuse agent can lead to ineffectiveness of the prophylactic agent and a need for close monitoring to improve outcomes [20,21].

Atogepant is a small-molecule CGRP receptor antagonist—a key neuropeptide in migraine pathophysiology—used in acute and preventive migraine treatment, reducing neurogenic inflammation and central sensitization [9]. In the current case, atogepant managed the MOH by providing effective preventive treatment, allowing triptan discontinuation. Clinical trials have demonstrated that gepants reduce the frequency of migraine days and are not associated with MOH. Their favorable safety profile and minimal side effects enhance patient adherence [22]. Providing both acute and prophylactic treatment, the CGRP class can replace traditional abortive therapies. Additionally, they are effective in managing chronic migraines associated with MOH, an outcome not achievable with other prophylactic agents [23].

On the other hand, stress has been identified as a significant trigger in 80% of patients with migraines. In the present case, stress was a major aggravating factor, causing headache relapse. Emotional strain, childcare demands, and sleep deprivation due to household responsibilities closely paralleled headache flares, reinforcing the role of non-pharmacologic management. Thus, incorporating behavioral therapy, relaxation training, and mindfulness can significantly improve outcomes when paired with pharmacologic therapy [24].

In the current case, addressing medication overuse was essential, as it worsened the headaches and reduced the effectiveness of preventive medications; thus, limiting acute medication use to less than 10–15 days per month is recommended. Erenumab reduces migraine frequency in certain cases; however, its efficacy in treating MOH is limited if triptan overuse persists [25] A meta-analysis found that triptans outperformed gepants for 2 h pain relief; however, they pose a higher risk of MOH and adverse events [26]. The symptom relapse during an atogepant shortage in the present case demonstrates the importance of continuous preventive access [27]. Moreover, unrestricted OTC triptan availability enabled recurrence even after education and prescription limitations, emphasizing the need for regulatory oversight of triptan use in Saudi Arabia [28]. CGRPs have demonstrated efficacy in reducing the frequency of migraine days in patients with chronic migraine [22], and they do not induce MOH; thus, they are ideal for patients overusing triptan [29]. Additionally, emerging evidence suggests that a once-daily oral preventive therapy may better align with patient preferences compared with monthly injectable treatments, particularly as the routine of daily dosing can provide psychological reassurance of continuous protection against potential acute migraine attacks [30]. Moreover, long-term safety data for CGRPs are promising, with no significant cardiovascular risks reported [22].

Although the availability of newer agents, such as gepants, represents an important advancement, the present case highlights that effective migraine management extends beyond pharmacotherapy alone. Researchers and clinicians often focus on developing or prescribing new medications, yet it remains essential to consider the patient as an individual with unique behavioral and psychosocial contributors to chronicity [31,32]. Interestingly, recent evidence suggests that migraine is frequently associated with cervical musculoskeletal impairments, temporomandibular dysfunction, myofascial pain, and altered pain processing, which may contribute to symptom persistence and disability. Recognition of these comorbid musculoskeletal factors is important because physiotherapy and multimodal rehabilitation interventions, including exercise therapy, manual therapy, and postural rehabilitation, have shown the potential to improve headache outcomes and functional status in selected patients [18,33]. An important aspect of this case was the patient’s active engagement in migraine self-management. As part of a comprehensive treatment approach, in addition to pharmacological therapy, the patient adopted stress-management strategies and physiotherapy exercises for the neck and upper back, which may have contributed to her sustained remission. Previous studies have shown that behavioral and self-management interventions can improve headache outcomes, reduce disability, and enhance patients’ ability to cope with migraine, particularly when combined with appropriate preventive treatment [34]. Moreover, this case highlights the important role of physicians in preventing MOH through patient education. Early counseling regarding the risks of frequent acute medication use, recognition of overuse patterns, and appropriate use of preventive therapies can reduce the risk of headache chronification and MOH [35]. Pharmacists can further identify excessive medication use and reinforce safe medication practice [36]. Documenting and publishing cases such as the present one can help broaden clinical and scientific awareness, encouraging practitioners to address non-pharmacological factors and guiding patients to manage these elements rather than relying exclusively on medication.

In conclusion, this case demonstrates the complexity of managing chronic migraine with MOH caused by triptan overuse. It highlights the critical role of family physicians in identifying modifiable factors and ensuring early diagnosis and appropriate management of chronic migraine. The patient’s lack of response to preventive therapies emphasizes the importance of addressing MOH through triptan withdrawal. The atogepant group targets both acute and preventive migraine needs, highlighting its therapeutic potential. However, triptan, while prone to overuse, is not associated with substance use disorder. Further research is required to optimize atogepant use and to regulate access to migraine drugs.

## Figures and Tables

**Table 1:** A timeline illustrating the clinical course of chronic migraine, treatment interventions, and therapeutic effectiveness.

Time Point	Preventive Therapy	Triptan Use Status	Duration of Therapy	Outcome/Status	Reason for Discontinuation or Change
Initial presentation	Propranolol 40 mg daily	Ongoing triptan use	4 years	Discontinued	Intolerable side effects
6 months later	Escitalopram added to propranolol	Continued triptan use	3 months	Discontinued	Planned switch to amitriptyline
3 months later	Amitriptyline	Continued triptan use	3 months	Discontinued	Weight gain, excessive sleepiness, and inadequate anxiety control
Subsequent 3 months	Escitalopram (for anxiety) + topiramate 25 mg twice daily	Continued triptan use	3 months	Discontinued	Inability to escalate dose due to intolerable side effects
Following 3 months	OnabotulinumtoxinA (Botox), 3 cycles	Continued triptan use	3 cycles	Discontinued	Lack of clinical effectiveness and inconsistent follow-up
Next 3 months	Erenumab	Triptan not available over the counter	6 months	Discontinued	Initial lack of improvement; partial benefit observed only during triptan unavailability
Most recent 6 months	Atogepant (Aquipta, 60 mg) + Ubrogepant (100 mg)	Daily triptan intake stopped	Ongoing	Continued	Effective headache control and suppression of compulsive daily medication use without significant adverse effects

## Data Availability

The data presented in this study are available on request from the corresponding author due to privacy.

## Abbreviations

The following abbreviations are used in this manuscript:

CGRP	calcitonin gene-related peptide
MOH	medication-overuse headache
OTC	over the counter
